# Supplementary material for: Separation and Determination of Chemopreventive Phytochemicals of Flavonoids from Brassicaceae Plants
Source: Molecules. 2021 Aug 5;26(16):4734. doi: 10.3390/molecules26164734 (PMC8399753; doi:10.3390/molecules26164734)
Supplement: Supplementary file 1 [file molecules-26-04734-s001.zip › molecules-1316681-supplementary.pdf]

**Table S1.** Content of the polyphenols in *Brassicaceae* plants extracts

| No sample | 1 | 2 | 3 | 4 | 5 | 6 | 7 | 8 | 9 | 10 | 11 | 12 | 13 | 14 | 15 | 16 | 17 | 18 | 19 | 20 | 21 | 22 | 23 | 24 | 25 |
|---|---|---|---|---|---|---|---|---|---|---|---|---|---|---|---|---|---|---|---|---|---|---|---|---|---|
| RUT | <LOD | <LOQ | <LOD | 178 | <LOQ | <LOQ | <LOD | <LOQ | <LOQ | <LOD | <LOD | 0.42 | <LOQ | 0.52 | <LOQ | <LOD | <LOD | <LOQ | <LOQ | <LOD | <LOQ | <LOQ | <LOD | <LOD | 0.74 |
| QUE | <LOQ | <LOQ | <LOQ | <LOQ | <LOQ | 1.05 | <LOQ | <LOQ | <LOQ | <LOQ | <LOQ | <LOQ | 0.94 | 0.92 | 1.56 | <LOQ | <LOQ | 61.19 | <LOQ | <LOQ | <LOQ | <LOQ | 20.9 | <LOQ | <LOQ |
| NAR | 1.05 | 0.65 | 1.88 | 0.55 | <LOQ | 0.40 | <LOQ | <LOQ | 0.86 | <LOQ | 0.67 | <LOQ | <LOQ | <LOQ | 2.00 | <LOQ | ND | <LOQ | <LOQ | <LOQ | ND | <LOQ | 1.31 | 0.82 | <LOQ |
| TAX | <LOQ | ND | ND | <LOQ | 0.52 | 0.42 | <LOQ | 0.44 | ND | ND | <LOQ | <LOQ | 0.41 | <LOQ | 1.15 | <LOQ | ND | 0.42 | 1.03 | <LOQ | ND | <LOQ | 3.39 | 0.41 | 0.58 |
| ERC | 4.46 | 3.53 | 1.81 | 6.54 | 0.71 | ND | ND | ND | ND | ND | ND | ND | ND | ND | ND | ND | ND | ND | ND | ND | ND | ND | ND | ND | ND |
| ERI | <LOQ | <LOQ | <LOQ | <LOQ | <LOQ | <LOQ | ND | 0.79 | <LOQ | <LOQ | <LOQ | <LOQ | <LOQ | 0.73 | 0.71 | ND | ND | ND | ND | <LOQ | <LOQ | ND | 0.97 | <LOQ | <LOQ |
| 3-HBA | 0.45 | 7.20 | 0.78 | 1.63 | 0.40 | <LOQ | <LOQ | <LOQ | 0.50 | <LOQ | <LOQ | <LOQ | <LOQ | <LOQ | <LOQ | <LOQ | <LOQ | 0.83 | 0.44 | <LOQ | <LOQ | 1.64 | 0.40 | <LOQ | 0.54 |
| BA | 0.64 | 1.32 | 2.98 | 2.30 | 0.61 | 2.03 | 1.24 | 3.23 | 0.87 | 1.44 | 2.21 | 0.82 | 0.99 | 0.67 | 2.43 | ND | ND | 1.78 | 1.23 | 1.73 | ND | ND | 0.63 | 1.01 | ND |
| CA | 0.66 | ND | ND | ND | ND | 0.40 | ND | ND | ND | ND | ND | ND | ND | ND | ND | ND | ND | ND | ND | ND | ND | ND | ND | ND | ND |
| 3,4-DHBA | 0.70 | 222 | 15.7 | 259 | 37.4 | 60.1 | 31.9 | 42.2 | 41.6 | 41.1 | 42.1 | 48.9 | 82.4 | 121 | 161 | 51.6 | 99.1 | 31.3 | 16.3 | 71.7 | 63.9 | 47.3 | 1167 | 61.9 | 45.3 |
| 4-HBA | 295 | 4054 | 1478 | 863 | 121 | 1516 | 291 | 619 | 25.5 | 139 | 349 | 222 | 142 | 495 | 462 | 72.7 | 53.2 | 317 | 270 | 1306 | 169 | 110 | 215 | 111 | 114 |
| 3,4-HPPA | 953 | 1346 | 868 | 65.3 | 75.5 | 92.9 | 70.7 | 36.5 | 527 | 194 | 123 | 122 | 93.8 | 51.2 | 103 | 106 | 504 | 705 | 127 | 94.4 | 74.9 | 182 | 121 | 143 | 517 |
| 3-HPA | ND | ND | ND | ND | ND | ND | ND | ND | ND | ND | ND | ND | ND | ND | ND | ND | ND | ND | ND | ND | ND | ND | ND | 2.18 | ND |
| *p*-COA | 248 | 804 | 71.9 | 1663 | 145 | 377 | 576 | 550 | 195 | 924 | 833 | 68.4 | 179 | 91.4 | 234 | 77.2 | 168 | 447 | 148 | 250 | 95.3 | 454 | 612 | 125 | 109 |
| FA | 10.2 | 5.17 | ND | ND | ND | ND | ND | ND | ND | ND | ND | ND | 0.51 | ND | ND | ND | ND | ND | ND | ND | ND | ND | 8.97 | ND | ND |
| DOPAC | ND | ND | ND | ND | ND | ND | ND | ND | ND | ND | ND | ND | ND | ND | ND | 0.53 | 0.40 | 0.66 | 0.91 | 1.21 | 0.40 | ND | ND | ND | ND |

α-HHA, HVA, HA, HST, HSD, NHSD, PIN, LQG, NRI, LIQ, FIS, NARG, GLB, FOR – not detected

1 – Radish – sprouts; 2 – Radish – roots; 3 – Radish – spots; 4 – Radish – leaves; 5 – Broccoli – sprouts; 6 – Broccoli – flowers; 7 – Brussels sprouts – leaves; 8 – Kale – leaves; 9 – Canola – roots; 10 – Canola – spots; 11 – Canola – leaves; 12 – Conehead cabbage – leaves; 13 – Kohlrabi – spots; 14 – Kohlrabi – edible spot; 15 – Kohlrabi – leaves; 16 – Green cabbage – leaves; 17 – Chinese cabbage – midvein; 18 – Chinese cabbage – leaves; 19 – Cauliflower – leaves; 20 – Cauliflower – head; 21 – Cauliflower – midvein; 22 – Pak Choi cabbage – leaves; 23 – Red cabbage – leaves; 24 – Italian cabbage – leaves; 25 – Kale – sprouts

**Table S2.** MRM transitions for selected polyphenols.

| Compound | *Q1*[a] (*m/z*) | *Q3*[b] (*m/z*) |
|---|---|---|
| **3,4-DHBA** | 152.9 | 108.9 |
| **α-HPA** | 193.9 | 72.8 |
| **DOPAC** | 166.9 | 122.7 |
| **4-HBA** | 136.9 | 93.0 |
| **CA** | 178.9 | 135.0 |
| **HA** | 177.9 | 133.7 |
| **3-HBA** | 136.9 | 92.6 |
| **3-HPA** | 150.9 | 107.0 |
| **HVA** | 180.8 | 136.8 |
| **3,4-HPPA** | 164.9 | 120.5 |
| **p-COA** | 162.9 | 119.2 |
| **FA** | 192.9 | 134.2 |
| **ERC** | 595.2 | 286.9 |
| **LIQ** | 417.2 | 255.0 |
| **RUT** | 609.0 | 299.7 |
| **TAX** | 303.2 | 284.7 |
| **BA** | 120.9 | 76.8 |
| **NRI** | 579.3 | 270.9 |
| **NARG** | 579.2 | 270.9 |
| **HSD** | 609.0 | 300.8 |
| **NHSD** | 609.0 | 300.8 |
| **FIS** | 284.9 | 134.8 |
| **LQG** | 255.1 | 118.7 |
| **ERI** | 287.0 | 150.7 |
| **QUE** | 300.9 | 150.7 |
| **NAR** | 270.9 | 150.7 |
| **HST** | 300.9 | 163.7 |
| **FOR** | 266.9 | 251.8 |
| **CHS (IS)** | 252.9 | 143.0 |
| **PIN** | 254.8 | 150.7 |
| **GLB** | 323.2 | 201.3 |

[a] Q1, precursor ion
[b] Q3, fragment ion
